# Effects of Inositol(s) in Women with PCOS: A Systematic Review of Randomized Controlled Trials

**DOI:** 10.1155/2016/1849162

**Published:** 2016-10-23

**Authors:** Vittorio Unfer, John E. Nestler, Zdravko A. Kamenov, Nikos Prapas, Fabio Facchinetti

**Affiliations:** ^1^Department of Medical Sciences, IPUS-Institute of Higher Education, Chiasso, Switzerland; ^2^Department of Medicine and Department of Obstetrics and Gynecology, Virginia Commonwealth University, Richmond, VA, USA; ^3^Clinic of Endocrinology, Alexandrovska University Hospital, Medical University, Sofia, Bulgaria; ^4^IAKENTRO, Infertility Treatment Center, Thessaloniki, Greece; ^5^Mother-Infant Department, University of Modena and Reggio Emilia, Modena, Italy

## Abstract

Polycystic ovary syndrome (PCOS) is a common endocrine disorder, with complex etiology and pathophysiology, which remains poorly understood. It affects about 5–10% of women of reproductive age who typically suffer from obesity, hyperandrogenism, ovarian dysfunction, and menstrual irregularity. Indeed, PCOS is the most common cause of anovulatory infertility in industrialized nations, and it is associated with insulin resistance, type 2 diabetes mellitus, and increased cardiovascular risk. Although insulin resistance is not included as a criterion for diagnosis, it is a critical pathological condition of PCOS. The purpose of this systematic review is the analysis of recent randomized clinical trials of inositol(s) in PCOS, in particular myo- and D-chiro-inositol, in order to better elucidate their physiological involvement in PCOS and potential therapeutic use, alone and in conjunction with assisted reproductive technologies, in the clinical treatment of women with PCOS.

## 1. Introduction

Polycystic ovary syndrome (PCOS) is one of the most common endocrine disorders affecting women of reproductive age. PCOS is associated with a wide range of maladies, such as hormonal and metabolic impairments, ovarian dysfunction, and menstrual irregularity. According to the Rotterdam criteria developed in 2003, PCOS is diagnosed if two out of the three following features are met: chronic oligo- or anovulation, anatomically polycystic ovaries on ultrasonography, and clinical and/or biochemical hyperandrogenism [1]. Although not included as criteria, insulin resistance and hyperinsulinemia are important etiologic factors associated with the typical clinical signs and hormonal disorders of PCOS. Indeed, insulin resistance along with hyperinsulinemia affects approximately 40–50% of PCOS patients, both lean and obese [2–6]; however, in obese women with PCOS the prevalence of insulin resistance accompanied by compensatory hyperinsulinemia approaches 80% [7]. Treatment of PCOS with insulin-sensitizing drugs, such as metformin, troglitazone, and pioglitazone, has been shown to improve ovulatory function and reduce circulating androgens, corroborating the critical link between insulin resistance and the pathogenesis of this syndrome. Of these insulin-sensitizing agents, metformin is most commonly used in the treatment of PCOS, although it has no official indication outside of type 2 diabetes in many countries and therefore it is considered as an off-label product when used in nondiabetic women with PCOS. Nevertheless, nausea, diarrhea, and weight increase are side effects of metformin, which reduce patients' compliance and the suitability of its use [3, 8, 9].

In the past two decades, several studies have reported the effectiveness of inositol(s), mainly the two stereoisomers myo-inositol (Myo-Ins) and D-chiro-inositol (D-chiro-Ins), in improving the pathological conditions associated with PCOS [3, 8–14]. Indeed, Myo-Ins and D-chiro-Ins have been shown to play different roles in the physiology and treatment of PCOS [15]. In the ovary, D-chiro-Ins is involved in insulin-mediated androgen synthesis [16], whereas Myo-Ins mediates glucose uptake and follicle stimulating hormone (FSH) signaling [14, 15, 17, 18]. In human ovaries, 99% of the intracellular pool of inositol consists of Myo-Ins and the remaining part consists of D-chiro-Ins [17]; imbalance of ovarian Myo-Ins and D-chiro-Ins concentrations, like a putative Myo-Ins deficiency, might impair the FSH signaling, as observed in PCOS patients [17–19]. D-chiro-Ins is synthetized from Myo-Ins through the epimerase enzyme, which in turn is stimulated by insulin [19]. The epimerase activity is increased in the theca cells, causing a deficit of Myo-Ins [19] and this appears to be a critical factor in the pathogenesis of PCOS. Indeed, reduced intraovarian Myo-Ins may adversely affect glucose uptake and metabolism of both oocytes and follicular cells. Since oocytes are characterized by high glucose consumption this would compromise oocyte quality.

Several studies have emphasized the pivotal role of Myo-Ins in improving oocyte quality [10, 14, 25, 31, 32]. Myo-Ins and D-chiro-Ins are intracellularly incorporated into inositol phosphoglycans (IPGs), which are second messengers of insulin, and some actions of insulin are mediated by these IPG mediators. A number of studies have suggested that insulin pathway impairment could be due to dysregulation of the IPG second messenger system [33, 34]. This is consonant with the studies of Nestler et al. which suggest that altered metabolism of inositol or IPG mediators contribute to the insulin resistance of women with PCOS [13]. Indeed, they have demonstrated that D-chiro-Ins supplementation replenished stores of the mediator and improved insulin sensitivity in both lean and obese women with PCOS [12, 13].

Given the physiologic role of inositol(s) in oocyte and spermatozoa development, the 2013 Florence International Consensus Conference on myo- and D-chiro-inositol in obstetrics and gynecology addressed the use of inositol(s) in assisted reproductive technologies (ART) [35]. In addition, a previous systematic review by Unfer et al. provided an overview of the clinical outcomes of Myo-Ins as a treatment to improve ovarian function, as well as hormonal and metabolic parameters, in PCOS women [14]. In the present systematic review, we present updated information about inositol(s), in particular Myo-Ins and D-chiro-Ins, through an analysis of recently published reports, in order to better outline the physiological involvement and clinical use of inositol(s) in PCOS and ART.

## 2. Methods

A critical review of the literature was performed by searching core databases to select pertinent scientific articles: Medline, Amed, and the Cochrane Library. We conducted a search over the period from January 1999 to May 2016, and only randomized controlled trials (RCTs), involving women with PCOS, were included in the present study. Search terms included “inositol,” “myo-inositol,” “D-chiro-inositol,” “polycystic ovary syndrome,” “oocyte quality,” “ovarian stimulation,” “in vitro fertilization,” “ovarian function,” and “insulin resistance.” No language restrictions were imposed. Data from treatments with Myo-Ins or D-chiro-Ins in combination with other drugs, as well as animal and in vitro investigations, were excluded. Full articles were obtained through either our own library or interlibrary loan, for all published studies that were considered eligible for inclusion in the review. As described below, a total of 12 studies were finally included for review.

The main outcomes we aimed to focus on were the following: glucose and insulin sensitivity, 17*β*-estradiol (E2), testosterone (T), androstenedione (A), the homeostatic model assessment (HOMA) index, sex hormone binding globulin (SHGB), r-FSH, stimulation days, oocyte quality, embryo quality, biochemical pregnancies, and pregnancy rate.

## 3. Results of the Literature Search

The systematic search yielded 102 papers for consideration. A total of 69 studies were excluded during the screening phase as not being pertinent. Of the remaining 33 studies, 21 did not meet the selection criteria. This left 12 studies that were included and analyzed in the final review (Tables 1, 2, 3, and 4). All the RCTs analyzed in this review studied patients with PCOS.

Eight trials evaluated the effect of Myo-Ins administration on hormonal levels and oocyte quality [10, 11, 20, 21, 25–27, 29]. In one trial, the effects of different concentrations of D-chiro-Ins on the oocytes quality were assessed [28]. Three RCTs evaluated the effects of combined therapy with Myo-Ins and D-chiro-Ins on oocyte quality and in vitro fertilization (IVF) outcomes [22, 23, 30].

Of note, two trials were randomized controlled Myo-Ins versus folic acid, as placebo [20, 25]; three were double-blind randomized controlled trial Myo-Ins versus folic acid [11, 21, 26]; one was a randomized controlled Myo-Ins versus metformin [27]. One study was a dose-response study of D-chiro-Ins on ovaries [28]. A single study, RCT, also compared the efficacy between Myo-Ins and D-chiro-Ins in improving oocyte quality [29]. In the last 3 RCTs, the combination of Myo-Ins/D-chiro-Ins (40 : 1) was examined in PCOS patients [22, 23, 30].

In the report of Genazzani et al. [20], PCOS patients were recruited in the trial and treated with either Myo-Ins plus folic acid (Inofolic®, LO.LI. Pharma, Rome, Italy) or folic acid alone (Table 1). The endocrine profile was evaluated and main outcomes are shown in Table 3. Consistent and significant changes were observed in the group receiving Myo-Ins plus folic acid. Indeed, prolactin (PRL), plasma luteinizing hormone (LH), and follicle stimulating hormone (FSH) ratio significantly decreased. The index of insulin sensitivity, expressed as glucose-to-insulin ratio, significantly increaed. The Ferriman-Gallwey score decreased after 12 weeks of Myo-Ins administration although the reduction was not statistically significant (22.7 ± 1.4 to 18.0 ± 0.8) whereas the reduction of the ovaries volumes was significant (12.2 ± 0.6 mL to 8.7 ± 0.8 mL, *p* < 0.05).

The study design and the endocrine profile after treatment obtained in the RCT of Costantino et al. [21] are shown in Tables 1 and 3. During the present study, a reduction in the systolic and diastolic blood pressure (SBP and DBP) values was observed in patients treated with Myo-Ins (131 ± 2 mmHg to 127 ± 2 mmHg and 88 ± 1 mmHg to 82 ± 3 mmHg, resp.), while these values increased in placebo group (128 ± 1 mmHg to 130 ± 1 mmHg, *p* = 0.002, and 86 ± 7 mmHg to 90 ± 1 mmHg, *p* = 0.001, resp.). Furthermore, in Myo-Ins group, plasma triglycerides decreased from 195 ± 20 mg/dL to 95 ± 17 mg/dL and total cholesterol significantly decreased from 210 ± 10 mg/dL to 171 ± 11 mg/dL. In Myo-Ins-treated group the composite whole body insulin sensitivity index (ISI) increased significantly from 2.80 ± 0.35 mg/dL to 5.05 ± 0.59 mg/dL, while it did not change in placebo group. Ovulation was restored in 69.5% of women in Myo-Ins group and 21% of placebo (*p* = 0.001). After treatment, the peak level of progesterone (P) was higher in Myo-Ins patients (15.1 ± 2.2 ng/mL) compared to placebo. Furthermore, there was a significant reduction of more than 50% in the serum dehydroepiandrosterone sulphate in Myo-Ins women (366 ± 47 *μ*g/dL to 188 ± 24 *μ*g/dL; *p* = 0.003), whereas it was not significant in placebo.

Gerli et al. [10, 11] evaluated the effect of Myo-Ins on ovarian and metabolic factors in PCOS subjects, in 2 different studies conducted in 2003 and 2007 (Tables 1 and 3); in the first trial [10], the ovulation frequency was significantly higher (*p* < 0.01) in Myo-Ins-treated group (23%) compared with placebo (13%). The main outcomes are defined in Table 3. In addition, it was found that E2 concentration increased only in Myo-Ins group during the first week of treatment inducing follicular maturation. The body mass index (BMI) and leptin were significantly reduced in treated patients, whereas body weight augmented in placebo. A significant increase in circulating high-density lipoprotein (HDL) was recorded in Myo-Ins women. In the second study [11], in addition to the main findings shown in Table 3, a significant increment of the ovulation frequency in Myo-Ins group compared to placebo was observed. All patients started treatment outside the luteal phase, and the delay to the first ovulation after starting the program was significantly shorter in the study group (24.5 versus 40.5, *p* = 0.02). The analysis on the first and eighth day of treatment showed that the Myo-Ins-treated group had a significant increase in E2 levels (*p* = 0.03), whereas controls showed no change. Circulating levels of inhibin B remained unvaried. Circulating leptin concentration declined in Myo-Ins patients, in contrast to controls. The low-density lipoprotein (LDL) showed a trend toward reduction, and the HDL increased significantly in Myo-Ins group.

In Nordio and Proietti study [22], the combination of Myo-Ins and D-chiro-Ins versus Myo-Ins alone was evaluated (Tables 1 and 3). Either treatment was efficacious in improving the ovulation function and metabolic parameters. Besides the main findings displayed in Table 3, a reduction of SBP and SDB was observed in both groups (Myo-Ins plus D-chiro-Ins, 131.0 ± 1.6 mmHg to 128.0 ± 1.2 mmHg and 88.0 ± 3.3 to 80 ± 2 mmHg, resp., versus Myo-Ins, 129.0 ± 2.5 mmHg to 127 ± 2 mmHg and 87.0 ± 2.6 mmHg to 82 ± 1 mmHg, resp.). Also BMI and waist-to-hip ratio (WHR) were reduced after treatment but not significantly.

In a very recent study [23], an improvement of patients' insulin resistance and ovulatory function was observed after Myo-Ins and D-chiro-Ins treatment, significantly rebalancing their endocrine and metabolic profiles (Tables 1 and 3).

Papaleo et al. [25] broaden the clinical use of Myo-Ins by evaluating its effect on oocyte quality and the ovarian stimulation protocol for PCOS women (Table 2). As can be seen in Table 4, the number of oocytes retrieved did not differ between the two groups, whereas in the group treated with Myo-Ins the number of immature oocytes and degenerated oocytes was significantly reduced (1.0 ± 0.9 versus 1.6 ± 1.0; *p* = 0.01), with a trend for increased percentage of metaphase II stage oocytes.

In the study of Ciotta et al. [26], oocyte's quality was assessed after the oocyte pick-up during the assisted reproductive technology (ART) procedure in women with PCOS (Table 2). Besides results shown in Table 4, the number of immature oocytes resulting significantly reduced in Myo-Ins group (degenerated oocytes 0.93% versus 14.37%, *p* < 0.02; germinal vesicles 1.4% versus 9.37%, *p* < 0.02) and the mean number of transferred embryos was significantly higher.

Raffone et al. [27] compared the effects of metformin (Glucophage®, Merck Pharma) and Myo-Ins (Inofolic, LO.LI. Pharma, Rome, Italy) on PCOS patients (Tables 2 and 4). In Myo-Ins group 65% of patients versus 50% of metformin group restored spontaneous ovulation activity, after a mean of 14.8 (±1.8) days and 16.7 (±2.5) days from day 1 of the menstrual cycle, respectively.

Fifty-four women diagnosed with PCOS were selected in the study of Isabella and Raffone, 2012 [28] (Table 2). Patients were randomized into 5 groups, including a placebo group and 4 groups that received 300, 600, 1200, and 2400 mg of D-chiro-Ins (Interquim s.a., Barcelona, Spain) daily, respectively. In addition to the main results reported in Table 4, they found that high D-chiro-Ins concentrations progressively increase the number of immature oocytes, in a significant manner (*p* < 0.04).

As shown in Tables 2 and 4, Unfer et al. [29] compared the efficacy of Myo-Ins and D-chiro-Ins in patients diagnosed with PCOS. The selected ones were randomly divided into two groups receiving either Myo-Ins or D-chiro-Ins (Table 2). Along with the main findings presented in Table 4, it was reported that the number of immature oocytes was significantly lower in Myo-Ins group compared to D-chiro-Ins group (0.69 ± 0.64 versus 2.23 ± 0.85; *p* < 0.01).

The combination 40 : 1 of Myo-Ins and D-chiro-Ins (Inofolic Combi, LO.LI. Pharma, Rome, Italy; patented) was also evaluated by Colazingari et al. [30], in PCOS patients undergoing IVF (Table 2). In this study, for evaluation of results, women age was also taken into account, dividing them into 2 further categories: ≤35 or >35 years. The combination of Myo-Ins and D-chiro-Ins gave a greater result in the ovarian stimulation protocol compared to D-chiro-Ins alone (Table 4). In Myo-Ins plus D-chiro-Ins patients, oocytes of high quality resulted and the number of degenerated oocytes was lower. In particular results showed that Myo-Ins plus D-chiro-Ins treatment reduced the number of degenerated oocytes in both age groups (≤35 years old: 1.04 ± 1.15 versus 1.82 ± 1.55; >35 years old: 1.00 ± 0.91 versus 1.45 ± 0.89).

## 4. Discussion

A critical review of the 12 RCTs included in this systematic review highlights that oral administration of Myo-Ins, alone or in combination with D-chiro-Ins, is capable of restoring spontaneous ovulation and improving fertility in women with PCOS.

Myo-Ins and D-chiro-Ins are 2 of the 9 different stereoisomers of inositol, polyol found in many foods, in particular cereals, nuts, and fruits as well as in human cells. They exert important actions in the control of glucose homeostasis and, when incorporated into phosphoglycans, have been shown to serve as second messengers involved in the signaling-transduction cascade of insulin [36, 37]; Myo-Ins and D-chiro-Ins are also involved in a number of biochemical pathways within oocytes [38, 39]. PCOS women have lower serum D-chiro-Ins levels and elevated urinary loss of D-chiro-Ins-IPG [40]. As noted above, inositol phosphoglycans (IPGs) are potentially important putative intracellular mediators of insulin action. It has been demonstrated that, in patients affected by PCOS, the metabolism of inositol is dysregulated, highlighting the subtle connection between insulin resistance and inositol deficiency in PCOS patients [41]. Indeed, in women with PCOS, insulin resistance and compensatory hyperinsulinemia due to dysregulation of inositol metabolism may actually be the major underlying cause of the disorder. Various studies have shown the role of D-chiro-Ins at low dosage in increasing insulin sensitivity and ovulation frequency, as well as in decreasing levels of lipid biomarkers and serum androgen [12, 13]. D-chiro-Ins is converted from Myo-Ins through insulin-stimulated NAD-dependent epimerase. Myo-Ins is the most abundant inositol isomer within the ovary, as suggested by the fact that approximately 99% of the ovarian intracellular pool of inositol consists of Myo-Ins [17]. Indeed, it was shown that an increased activity of epimerase in theca cells of ovaries of PCOS women is associated with a consistent reduction in the intraovarian ratio of Myo-Ins to D-chiro-Ins [19]. These experimental data are in line with the so-called D-chiro-Ins ovarian paradox posited by Carlomagno et al. [18]; these investigators advanced the hypothesis that epimerase activity is increased in the ovaries of PCOS subjects, resulting in a local Myo-Ins deficiency responsible for the oligoovulation and poor oocyte quality of the disorder. This hypothesis has drawn attention to the importance of Myo-Ins and D-chiro-Ins supplementation in a physiological ratio in order to restore normal ovary functionality. In fact, a correlation between Myo-Ins concentration in the follicular fluid and high oocyte quality was found and a number of studies have reported that Myo-Ins supplementation is able to improve oocyte quality [25, 31].

In this systematic review a number of recent articles were selected in order to critically analyze the roles of Myo-Ins and D-chiro-Ins, combined or alone, as a treatment of PCOS. Although there are a number of published articles on the use of Myo-Ins as a treatment in women with PCOS, only few of them were designed as RCT. These RCT studies, reviewed here, support the hypothesis of a primary role of IPGs as second messengers of insulin signaling and demonstrate that Myo-Ins supplementation beneficially affects the hormonal milieu of PCOS patients. Indeed, these trials provide evidence that Myo-Ins reduces insulin levels, probably either by conversion to D-chiro-Ins (via the epimerase enzyme) or by serving as substrate for the formation of Myo-Ins-containing IPGs and D-chiro-Ins-containing IPGs, which would in turn amplify insulin signaling. In particular, two studies [20, 25] suggest that deficiency of Myo-Ins and/or D-chiro-Ins might be an additional cofactor contributing to the pathophysiology of the insulin resistance of PCOS patients [42]. In these studies, hormonal parameters improved significantly in all PCOS patients treated with Myo-Ins [10, 11, 20, 21, 25–27, 29]. In a study by Gerli et al. body weight and circulating leptin decreased significantly and HDL concentrations increased significantly in the patients treated with Myo-Ins, compared with the placebo group, providing the first indication that Myo-Ins treatment might possibly reduce the risk of cardiovascular diseases in PCOS women. Moreover, in an equivalency study, Raffone et al. [27] stated that Myo-Ins improves the pregnancy rate in PCOS women. These findings further support the hypothesis of a key role of IPG as second messenger of insulin signaling. The oral supplementation of Myo-Ins might reduce insulin levels, by providing a higher availability of IPG precursors, in this way improving the activities of this second messenger of insulin signal [27].

The study by Ciotta et al. demonstrated that Myo-Ins treatment reduced the number of germinal vesicles and degenerated oocytes and improved the development of mature oocytes, as previously reported in experimental data [43]. The authors concluded that Myo-Ins alone is useful in PCOS patients as insulin-sensitizer and for induction of oocyte maturation [26], confirming that Myo-Ins is likely an important constituent of the follicular microenvironment for normal nuclear and cytoplasmic oocyte's development.

As already noted, the role played by D-chiro-Ins in ovarian physiology is controversial. In this regard, a study in which different concentrations of D-chiro-Ins were administrated to nonobese PCOS women with normal insulin sensitivity undergoing IVF reported that as the dosage of D-chiro-Ins was progressively increased, oocyte quality and ovarian response worsened [28]. A possible explanation for this observation may lie in the different tissue-specific ratios of Myo-Ins/D-chiro-Ins in different organs (i.e., 100 : 1 in the ovary) and the diverse physiological roles of inositol stereoisomers, as Myo-Ins increases glucose cellular uptake and D-chiro-Ins is involved in glycogen synthesis [33, 44]. In fact, cells responsible for glycogen storage (such as liver, muscles, and fat cells) contain high levels of D-chiro-Ins, whereas brain and heart cells contain high concentration of Myo-Ins, since they require high consumption of glucose. These data are in line with the D-chiro-Ins paradox hypothesis and with the data of Unfer et al. (2011) that demonstrated that Myo-Ins rather than D-chiro-Ins improved oocyte quality in intracytoplasmic sperm injection cycles [18, 29]. To wit, Unfer et al. demonstrated that Myo-Ins treatment significantly reduced ovarian stimulation days and the IU of r-FSH administrated and improved both oocyte and embryo quality in euglycemic PCOS patients when compared with treatment with D-chiro-Ins. This was also shown in 2009 by Papaleo et al. and included in our previous systematic review [14, 25]. However, as demonstrated by Nordio and Proietti, the combination of Myo-Ins and D-chiro-Ins, at a physiological ratio of 40 : 1, was able to more quickly restore to normal the hormonal and metabolic parameters in overweight PCOS women than Myo-Ins treatment alone [22]. Bearing in mind previous studies, the physiological ratio of these two isomers (40 : 1) seems to be an optimal and promising approach for the treatment of PCOS disorders [45, 46].

This might be due to the synergistic action of Myo-Ins and D-chiro-Ins, as they regulate different biological processes. In fact, the combination of Myo-Ins and D-chiro-Ins may be particularly beneficial in overweight PCOS women, considering that Myo-Ins improves the ovulatory function and D-chiro-Ins rapidly reduces the peripheral hyperinsulinemia. Notably, Colazingari et al. also reported that combined therapy of Myo-Ins and D-chiro-Ins, rather than D-chiro-Ins alone, improved oocyte quality in PCOS women undergoing ART [30]. This study further corroborates previous data, suggesting that D-chiro-Ins supplementation alone might not be the optimal or appropriate approach for improving IVF outcomes in PCOS patients.

Treatment with the combination of Myo-Ins and D-chiro-Ins has been further investigated by Benelli et al. who demonstrated that these two molecules, together in a 40 : 1 ratio, improved the endocrine profile and insulin resistance of obese women with PCOS [23]. An important aspect of this study was that no relevant side effects were recorded during combined therapy with Myo-Ins and D-chiro-Ins, providing further evidence of the safety of the usage of these two stereoisomers in combination. There is also accumulating evidence on the beneficial effects of Myo-Ins administration on reproductive function and the efficacy of combined Myo-Ins/D-chiro-Ins administration, in the physiological plasma ratio of 40 : 1, for amelioration of the metabolic aberrations of PCOS and for restoring spontaneous ovulation [47].

In conclusion, the analysis of these clinical trials highlights the salutary effects of Myo-Ins supplementation in improving several of the hormonal and reproductive disturbances of PCOS; furthermore, the analysis lends prominence to the pivotal role of inositol(s), mainly Myo-Ins and D-chiro-Ins, as a safe and effective therapy for PCOS, including an enhanced oocyte follicular development and oocyte maturation and in stimulation and pregnancy outcomes in IVF procedures.

## Figures and Tables

**Table 1:** Eligible RCTs where myo-inositol and/or D-chiro-inositol have been evaluated for the treatment of PCOS patients.

Ref	Study design	Duration	Treatment	Number of subjects	Inclusion criteria	Exclusion criteria	Assessment of the response
[20]	Randomized, controlled	12 weeks	Treated group: Myo-Ins 2 g + FA 200 *µ*g/d Control group: FA 200 *µ*g/d	Number = 20 Treated: 10 Control: 10	PCOS, oligo/amenorrhea, normal PRL levels (range 5–25 ng/mL), and mild to severe hirsutism and/or acne	Hormone treatments in the last 24 weeks; adrenal enzymatic deficiency and/or other endocrine diseases	LH, FSH, PRL, E2, A, 17OHP, T, insulin, cortisol, OGTT^a^ for insulin, glucose, C-peptide determinations, vaginal ultrasound examination Ferriman-Gallwey score, BMI, and HOMA index

[21]	Double-blind, randomized, controlled	12–16 weeks	Treated group: Myo-Ins 4 g + FA 400 *µ*g/d Control group: FA 400 *µ*g/d	Number = 42 Treated: 23 Control: 19	Age: <40 years PCOS, oligomenorrhea, and high serum-free T and/or hirsutism	Not described	Systolic/diastolic blood pressure, triglycerides, cholesterol, BMI, WHR, plasma glucose and insulin sensitivity, total/free T, DHEAS, SHBG, A, and progesterone peak value

[10]	Double-blind, randomized, controlled	16 weeks	Treated group: Myo-Ins 200 mg + FA 800 *µ*g/d Control group: matching placebo	Number = 283 Treated: 136 Control: 147	Age: <35 years PCOS according to Adams et al. criteria^b^, oligo/amenorrhea	Hyperprolactinemia, abnormal thyroid function tests, and congenital adrenal hyperplasia	E2, P and LH, BMI, ovulation frequency, inhibin-b, fasting glucose, fasting insulin, or insulin AUC, VLDL, LDL, HDL, total cholesterol, and triglycerides

[11]	Double-blind, randomized, controlled	16 weeks	Treated group: Myo-Ins 4 g + FA 400 *µ*g/d Control group: FA 400 *µ*g/d	Number = 92 Treated: 45 Control: 47	Age: <35 years PCOS according to Adams et al. criteria^b^, oligo/amenorrhea	Hyperprolactinemia, abnormal thyroid function tests, and congenital adrenal hyperplasia	E2, P and LH, ratio of luteal phase weeks to observation weeks; inhibin-b, fasting glucose, fasting insulin, or insulin AUC, VLDL, LDL, HDL, total cholesterol, BMI, and triglycerides

[22]	Randomized controlled	24 weeks	Treated group: Myo-Ins 1.1 g + D-chiro-Ins 27.6 mg/d Control group: Myo-Ins 4 g/d	Number = 50 Treated: 26 Control: 24	Age: <41 years, BMI >27 kg/m^2^, and PCOS according to Rotterdam criteria	Diabetic subjects, smokers, and alcohol users	Blood pressure, BMI, WHR, SHBG, serum steroids and lipid profile levels, OGTT, plasma glucose insulin, HOMA, and P

[23]	Randomized controlled	24 weeks	Treated group: Myo-Ins 1.1 g + D-chiro-Ins 27.6 mg + FA 400 *μ*g/d Control group: FA 400 *μ*g/d	Number = 46 Treated: 21 Control: 25	Age: <35 years, BMI >30 kg/m^2^, and PCOS according to Rotterdam criteria	Diabetic subjects, smokers, and alcohol users	FSH, LH, E2, SHBG, A, free T, DHEA-S, HOMA index, and fasting glucose and insulin

Myo-Ins, myo-inositol; D-chiro-Ins, D-chiro-inositol; FA, folic acid; PCOS, polycystic ovary syndrome; PRL, prolactin; E2, oestradiol; A, androstenedione; 17OHP, 17-hydroxyprogesterone; T, testosterone; P, progesterone; OGTT, oral glucose tolerance; BMI, body mass index; LH, luteinizing hormone; FSH, follicle stimulating hormone; DHEAS, dehydroepiandrosterone; SHBG, sex hormone binding globulin; AUC, area under the curve of OGTT; VLDL, very-low-density lipoprotein; LDL, low-density lipoprotein; HDL, high-density lipoprotein; WHR, waist-to-hip ratio.

^a^OGTT performed sampling 15 minutes before and 30, 60, 90, 120, and 240 minutes after the oral assumption of 75 g of glucose.

^b^Adams et al. [24].

**Table 2:** Eligible RCTs where myo-inositol and/or D-chiro-inositol have been evaluated for the treatment of PCOS patients undergoing ART.

Ref	Study design	Duration	Treatment	Number of subjects	Inclusion criteria	Exclusion criteria	Assessment of the response
[25]	Randomized, controlled	During ovulation induction for ICSI	Treated group: Myo-Ins 4 g + FA 400 *µ*g/d Control group: FA 400 *µ*g/d	Number = 60 Treated: 30 Control: 30	Age: <40 years, PCOS, oligo/amenorrhea, hyperandrogenism, hyperandrogenemia, typical features of ovaries on ultrasound scan	Hyperinsulinemia, hyperprolactinemia, hypothyroidism, androgen excess due to adrenal hyperplasia or Cushing syndrome	E2, stimulation (days), FSH IU Number of retrieved oocytes Number of MII, number of immature oocytes Number of embryos grade 1, embryo cleavage rate, fertilization rate Number of biochemical pregnancies Number of abortion cancellation rate Ovarian hyperstimulation syndrome

[26]	Double-blind randomized controlled	12 weeks	Treated group: Myo-Ins 4 g + FA 400 *μ*g/d Control group: FA 400 *μ*g/d	Number = 34 Treated: 17 Control: 17	Age: <40 years, PCOS, oligo/amenorrhea, hyperandrogenism, hyperandrogenemia, typical features of ovaries on ultrasound scan	Hypothyroidism, hyperthyroidism, diabetes mellitus, androgen-secreting cancers, adrenal hyperplasia, Cushing syndrome	E2, total r-FSH Number of follicles with a diameter >15 mm Number of oocytes retrieved Number of immature oocytes Number of embryos grade 1 Number of transferred embryos Number of biochemical pregnancies

[27]	Randomized, controlled	24 weeks	Treated group: Myo-Ins 4 g + FA 400 *µ*g/d Control group: metformin 1.5 g/d	Number = 120 Treated: 60 Control: 60	Age: <35 years, PCOS according to Rotterdam criteria	Hyperprolactinemia, hypothyroidism, androgen excess, adrenal hyperplasia or Cushing's syndrome, tubal defects, semen parameters defects	Restoration of spontaneous ovarian activity by weekly serum P dosage and a transvaginal ultrasound scan documenting the presence of follicular growth or luteal cyst Number of pregnancies Abortion rate

[28]	Randomized controlled	8 weeks before r-FSH	Treated group: D-chiro-Ins 300, 600, 1200, and 2400 mg/d Control group: placebo	Number = 54 Treated: 4 groups (10–12 pts) Control: 11	Age: <40 years, PCOS according to Rotterdam criteria, undergoing ICSI procedure	Insulin resistance and/or hyperglycaemia	Total r-FSH, E2, stimulation (days) Number of oocytes retrieved Number of cycles cancelled Number of MII, number of immature oocytes Number of embryos grade 1

[29]	Randomized controlled	8 weeks before r-FSH	Treated group: Myo-Ins 4 g/d Control group: D-chiro-Ins 1.2 g/d	Number = 84 Treated: 43 Control: 41	Age: <40 years, PCOS according to Rotterdam criteria, undergoing ICSI procedure	Insulin resistance and/or hyperglycaemia	Duration of infertility, BMI, PRL, TSH, E2, stimulation (days), FSH Number of cancelled cycles Number of retrieved oocytes Number of MII, number of immature oocytes Number of embryos grade 1 Number of biochemical/clinical pregnancies Number of spontaneous abortions

[30]	Randomized controlled	12 weeks before r-FSH	Treated group: Myo-Ins 1.1 g + D-chiro-Ins 27.6 mg/d Control group: D-chiro-Ins 1 g/d	Number = 100 Treated: 47 Control: 53	Age: ≤35 years, >35 years BMI <28 kg/m^2^, FSH <10 IU/L PCOS according to Rotterdam 2003 and a normal uterine cavity	Advanced stage (III or IV) endometriosis Poor responders pts or suffering from premature ovarian failure	Total IU of r-FSH, E2 before hCG injection Number of MII, number of VG-DEG Number of embryos grade 1 Number of embryos transferred Maturation rate and fertilization rate

Myo-Ins, myo-inositol; D-chiro-Ins, D-chiro-inositol; FA, folic acid; PCOS, polycystic ovary syndrome; E2, oestradiol; r-FSH, recombinant follicle stimulating hormone; MII, mature oocytes; VG-DEG, immature oocytes and degenerated oocytes; hCG, Human Chorionic Gonadotropin; ART, assisted reproductive technology.

**Table 3:** Biochemical and clinical findings related to hyperandrogenism and metabolism.

Ref	Treatment	Testosterone (ng/dL)	Androstenedione (ng/mL)	Free testosterone (ng/dL)	Insulin (*µ*U/mL)	HOMA index	OGTT^a^	SHBG (nmol/L)	General findings
[20]^c^	Myo-Ins versus FA	54.8 ± 6.2 versus 55.2 ± 9.1	1.70 ± 0.29 versus 1.91 ± 0.24	NA	6.5 ± 1.1^*∗∗∗*§§§^ versus 11.3 ± 1.1	1.4 ± 0.3^*∗∗*§§^ versus 2.5 ± 0.7	Myo-Ins improved glucose tolerance	NA	Myo-Ins significantly reduced LH, PRL, insulin levels, and LH/FSH ratio and significantly improved insulin sensitivity and menstrual cyclicity was restored in amenorrheic and oligomenorrheic subjects.

[21]^c^	Myo-Ins versus FA	34.8 ± 4.3^§§^ versus 109.0 ± 7.5	1.96 ± 0.26 versus 3.06 ± 0.41	0.24 ± 0.03^§§^ versus 0.85 ± 0.13	26.0 ± 8.0 versus 38.0 ± 7.0	NA	Myo-Ins improved glucose tolerance	198.0 ± 24.0 versus 163.0 ± 26.0	Myo-Ins increased insulin sensitivity and improved glucose tolerance and insulin release. There was a significant reduction in total and free T. There was a decrement in systolic and diastolic blood pressure. Plasma triglycerides and total cholesterol concentration decreased.

[10]^d^	Myo-Ins versus placebo	101.0 (81–121)^§^ versus 121.0 (101–141)	Decreased in Myo-Ins group	NA	No significant difference	NA	No significant difference	36.5^§^ versus 26.3	Myo-Ins showed a beneficial effect in improving ovarian function in PCOS women with oligomenorrhea.

[11]^d^	Myo-Ins versus FA	95.0 (72–115) versus 118.0 (98–138)	Decreased in Myo-Ins group	NA	16.8 versus 17.3	NA	No significant difference	35.9^§^ versus 25.8	Myo-Ins treatment showed a beneficial effect in improving ovarian function, anthropometric measures, and lipid profile.

[22]	Myo-Ins + D-chiro-Ins versus Myo-Ins	32.7 ± 10.0^*∗∗*^ versus 40.1 ± 9.5^*∗∗*^	1.94 ± 0.15^*∗∗*^ versus 1.98 ± 0.19^*∗∗*^	0.23 ± 0.02^*∗∗*^ versus 0.24 ± 0.03^*∗∗*^	9.2 ± 2.1^*∗∗*^ versus 9.6 ± 1.9^*∗∗*^	1.5 ± 0.28^*∗∗*^ versus 1.9 ± 2.1^*∗∗*^	Myo-Ins + D-chiro-Ins improved glucose tolerance	208 ± 20^*∗*^ versus 202 ± 27^*∗*^	Both treatments, Myo-Ins + D-chiro-Ins, or Myo-Ins alone normalized the metabolic parameters and restored ovulation in overweight PCOS women. At the end of the treatment both the fasting insulin and glucose serum concentration level were significantly reduced. However, compared to Myo-Ins alone, the combined treatment has shown significant changes on the metabolic profile after only 12 weeks.

[23]	Myo-Ins + D-chiro-Ins versus Myo-Ins	NA	4.01 ± 1.7 versus 3.12 ± 2.23	0.62 ± 0.15^*∗*^ versus 0.83 ± 0.2	10.7 ± 5.5^*∗∗∗*^ versus 17.8 ± 8.2	1.97 ± 1.48^*∗*^ versus 2.8 ± 1.4	NA	35.85 ± 24.3^*∗*^ versus 21.36 ± 7.57	Myo-Ins + D-chiro-Ins decreased significantly LH, free T levels, HOMA index, and fasting insulin. The combined treatment significantly increased E2 and SHBG. No relevant side effects were recorded. Therefore, the combined treatment, Myo-Ins + D-chiro-Ins, is effective in improving endocrine and metabolic parameters in young obese PCOS women.

Myo-Ins, myo-inositol; D-chiro-Ins, D-chiro-inositol; FA, folic acid; PCOS, polycystic ovary syndrome; PRL, prolactin; E2, oestradiol; A, androstenedione; 17OHP, 17-hydroxyprogesterone; T, testosterone; P, progesterone; OGTT, oral glucose tolerance; BMI, body mass index; LH, luteinizing hormone; FSH, follicle stimulating hormone; DHEAS, dehydroepiandrosterone; SHBG, sex hormone binding globulin; AUC, area under the curve of OGTT; VLDL, very-low-density lipoprotein; LDL, low-density lipoprotein; HDL, high-density lipoprotein; WHR, waist-to-hip ratio.

^a^OGTT performed sampling 15 minutes before and 30, 60, 90, 120, and 240 minutes after the oral assumption of 75 g of glucose.

Values are mean ± SD. ^c^Values are mean ± SEM. ^d^Values are mean (CIs), confidence intervals (95%). A brief description is inserted in the table when numerical data are not available in the original article. The units were made uniform to show more comparable results.

*p* value: ≤0.05^§^; ≤0.01^§§^; ≤0.001^§§§^: comparison posttreatment experimental group versus control.

*p* value: ≤0.05^*∗*^; ≤0.01^*∗∗*^; ≤0.001^*∗∗∗*^: comparison posttreatment with respect to baseline. Data at baseline are not shown in the table.

**Table 4:** IVF parameters and fertilization outcomes.

Ref	Treatment	E2 (pg/mL)		r-FSH dose (IU)	Stimulation days	MII	Oocyte retrieved	Embryo grade 1	Biochemical pregnancy (%)	Pregnancy rate (%)	General findings
[25]	Myo-Ins versus FA	2,232 ± 510^§^ versus 2,713 ± 595		1,958 ± 695^§^ versus 2,383 ± 578	11.4 ± 0.9^§^ versus 12.4 ± 1.4	7.14 ± 3.49 versus 7.07 ± 3.04	8.76 ± 4.12 versus 9.37 ± 3.31	0.86 ± 0.83 versus 0.81 ± 0.83	9.1 versus 10	14.6 versus 12.9	Myo-Ins significantly reduced E2 at hCG administration, total r-FSH units, number of stimulation days, and number of VG-DEG, with a trend for increased percentage of oocytes in MII. Number of oocytes retrieved did not differ in the 2 groups.

[26]^e^	Myo-Ins versus FA	Reduced in Myo-Ins group versus control		Reduced in Myo-Ins group versus control	NA	82.24% versus 66.87%	12^§^ versus 8.50	68.1%^§§^ versus 29%	No differences	NA	Myo-Ins has a positive effect on mature oocytes development and reduction of E2 and total r-FSH. Number of follicles with a diameter >15 mm visible at ultrasound scan during stimulation and the number of oocytes retrieved at the pick-up resulted significantly higher in the Myo-Ins-treated group. The number of immature oocytes was significantly reduced, and there was an increasing trend of the rate of oocytes in MII.

[27]	Myo-Ins versus metformin	NA		3 cycles × 37.5 U/day (if no pregnancy occurred)	NA	NA	NA	NA	30 versus 18.3	48.3 versus 36.6	Both Myo-Ins and metformin can be considered as first-line treatment for restoring normal menstrual cycles in most patients with PCOS; however Myo-Ins treatment seems to be more effective than metformin.

[28]	D-chiro-Ins (2400 mg) versus placebo	1,490.24 ± 253.21^§^ versus 1,429.69 ± 1,118.43		2,983.0 ± 219.80^§§^ versus 2,239.7 ± 181.55	13.8 ± 0.87^§§^ versus 11.4 ± 1.2	Decreased progressively after D-chiro-Ins administration	No differences	Decreased progressively after D-chiro-Ins administration	NA	NA	High D-chiro-Ins dosage negatively affects oocyte quality. It worsens oocyte quality and ovarian response in nonobese and non-insulin resistant PCOS women.

[29]	Myo-Ins versus D-chiro-Ins	2,261.2 ± 456.6^§§^ versus 2,740 ± 396.67		1,953.6 ± 397.5^§§^ versus 2,360.5 ± 301.9	11.1 ± 0.8^§§^ versus 12.7 ± 1.1	8.21 ± 2.39^§^ versus 7.08 ± 2.67	8.90 ± 2.84 versus 9.32 ± 3.15	1.64 ± 0.88^§§^ versus 0.76 ± 0.43	14 versus 9	51^§^ versus 24	Myo-Ins significantly increased number of MII and decreased number of immature oocytes compared to D-chiro-Ins. Furthermore, it increased the mean number of top quality embryos and the total number of pregnancies compared to D-chiro-Ins. Number of oocytes retrieved did not differ in the two treatments groups.

[30]	Myo-Ins + D-chiro-Ins versus D-chiro-Ins	Age ≤35	2,230.09 ± 827.57 versus 2,537.94 ± 860.19	1,569.02 ± 497.12^§^ versus 1,899.21 ± 618.17	NA	7.91 ± 4.51 versus 8.00 ± 3.92	9.91 ± 4.85 versus 10.79 ± 4.66	0.96 ± 0.83^§§§^ versus 0.73 ± 0.73	NA	NA	The combined treatment with Myo-Ins + D-chiro-Ins, rather than D-chiro-Ins alone, was able to improve oocyte quality and high-quality embryos in PCOS women undergoing ART regardless of the age.
		Age ≥35	2,185.09 ± 409.08^§^ versus 2,519.85 ± 788.49	1,906.96 ± 770.59 versus 2,170.58 ± 694.44	NA	6.91 ± 2.26 versus 8.35 ± 5.19	8.35 ± 3.21^§^ versus 10.75 ± 5.23	0.90 ± 0.80^§^ versus 0.68 ± 0.80	NA	NA	

Myo-Ins, myo-inositol; D-chiro-Ins, D-chiro-inositol; FA, folic acid; PCOS, polycystic ovary syndrome; E2, oestradiol; r-FSH, recombinant follicle stimulating hormone; MII, mature oocytes; VG-DEG, immature oocytes and degenerated oocytes; hCG, Human Chorionic Gonadotropin; ART, assisted reproductive technology.

Values are mean ± SD. ^e^Values are shown as median. A brief description is inserted in the table when numerical data are not available.

*p* value: ≤0.05^§^; ≤0.01^§§^; ≤0.001^§§§^: comparison posttreatment experimental group versus control.
